# Therapeutic Impact of Zanubrutinib in Chronic Lymphocytic Leukemia: Evidence from a Systematic Review and Single-Arm Meta-Analysis

**DOI:** 10.3390/ph18111674

**Published:** 2025-11-05

**Authors:** Yasser Alatawi, Fawaz E. Alanazi, Abdullah Alattar, Reem Alshaman, Yasmin N. Ramadan, Reem Sayad, Helal F. Hetta

**Affiliations:** 1 Department of Pharmacy Practice, Faculty of Pharmacy, University of Tabuk, Tabuk 71491, Saudi Arabia; yasser@ut.edu.sa; 2 Department of Pharmacology and Toxicology, Faculty of Pharmacy, University of Tabuk, Tabuk 71491, Saudi Arabia; falanazi@ut.edu.sa (F.E.A.); aalattar@ut.edu.sa (A.A.); ralshaman@ut.edu.sa (R.A.); 3 Department of Microbiology and Immunology, Faculty of Pharmacy, Assiut University, Assiut 71515, Egypt; yasmine_mohamed@pharm.aun.edu.eg; 4 Department of Histology, Faculty of Medicine, Assiut University, Assiut 71515, Egypt; reem.17289806@med.aun.edu.eg; 5 Division of Microbiology, Immunology and Biotechnology, Department of Natural Products and Alternative Medicine, Faculty of Pharmacy, University of Tabuk, Tabuk 71491, Saudi Arabia

**Keywords:** zanubrutinib, BGB-3111, Bruton’s tyrosine kinase inhibitor, chronic lymphocytic leukemia, small lymphocytic lymphoma, relapsed/refractory CLL, treatment-naïve

## Abstract

**Background and Objective:** Zanubrutinib, a next-generation Bruton’s tyrosine kinase inhibitor (BTKi), has demonstrated promising efficacy in chronic lymphocytic leukemia (CLL), including treatment-naïve (TN) and relapsed/refractory (R/R) patients. However, evidence synthesis across clinical trials remains limited. We conducted a systematic review and single-arm meta-analysis to evaluate the efficacy of zanubrutinib in CLL. **Methods:** This study was performed in accordance with PRISMA guidelines and Cochrane recommendations. PubMed, Medline, Scopus, and Web of Science were searched up to August 2025 using terms related to zanubrutinib and CLL/SLL. Eligible studies included clinical trials of zanubrutinib in TN or R/R CLL/SLL patients. Risk of bias was assessed using the JBI tool for non-randomized studies and for RCTs. Pooled estimates of efficacy outcomes were calculated using a random-effects model. Pooled estimates were calculated using the DerSimonian–Laird random-effects model, which accounts for both within- and between-study variability. **Results:** Seven studies (*n* > 1000) were included, enrolling both TN and R/R patients across diverse global populations. The pooled overall response rate (ORR) was 93.3% (95% CI, 86.7–99.8%) in mixed TN and R/R populations, 94.4% (95% CI, 91.6–97.3%) in TN patients, and 83.9% (95% CI, 75.0–92.8%) in R/R patients. Complete response (CR) rates were 12.2% (95% CI, 0.3–24.2%) overall, 13.8% (95% CI, 1.5–26.2%) in TN patients, and 5.0% (95% CI, 0.3–9.8%) in R/R patients. Partial response (PR) rates reached 86.0% (95% CI, 82.6–89.5%) in TN and 63.2% (95% CI, 53.5–73.0%) in R/R patients. Progressive disease was rare (≤1% in R/R cohorts). Heterogeneity was moderate to high across several outcomes. **Conclusions:** Zanubrutinib demonstrates favorable efficacy in CLL, achieving high ORR in both TN and R/R patients, with particularly durable responses in TN populations. Although complete response rates remain modest, especially among R/R patients, overall disease control appears consistent. These findings support zanubrutinib as an effective treatment option across CLL settings; however, variability among studies and the modest CR rates highlight the need for longer follow-up and direct comparative trials to further define its clinical role.

## 1. Introduction

Chronic lymphocytic leukemia and small lymphocytic lymphoma (CLL/SLL) are B-cell cancers that are incurable with traditional cytotoxic chemotherapy [1,2,3]. Over the past ten years, the landscape of CLL/SLL treatment has changed. One of the most recent developments in treatment paradigms is the advent of Bruton tyrosine kinase inhibitors (BTKis), which block the BTK enzyme, a crucial intracellular mediator of B-cell receptor signaling that supports the proliferation and survival of cancerous B cells [4,5,6].

Ibrutinib, an oral medication approved in Japan for the treatment of CLL, is a first-generation BTKi; however, it may be linked to treatment resistance and a higher incidence of cardiovascular adverse events (AEs), such as hypertension and atrial fibrillation [7]. Although BTKis have helped CLL patients receive better treatment outcomes, there is still a need for safer and more efficient alternatives.

Zanubrutinib is a potent, irreversible, selective, second-generation BTKi intended to optimize BTK occupancy while reducing off-target kinase inhibition [8]. In global phase 3 trials, zanubrutinib has shown superior safety and efficacy versus bendamustine plus rituximab in patients with treatment-naive (TN) CLL/SLL [9] and versus ibrutinib in patients with relapsed/refractory (R/R) CLL/SLL [10]. In the US [11], Europe [12], and more than 70 other countries, zanubrutinib is authorized for these uses. A multicenter, open-label, phase 1/2 BGB-3111-111 trial is the first investigation into the safety and effectiveness of zanubrutinib in Japanese patients with treatment-naïve (TN) CLL/SLL and R/R CLL/SLL [13]. This trial demonstrated that zanubrutinib was both effective and tolerable for Japanese patients; all cohorts had a high overall response rate (ORRs) (92–100%) [13]. Numerous international trials, including three phase III trials (SEQUOIA, ALPINE, and ASPEN), have demonstrated zanubrutinib’s excellent effectiveness and sustained response in various patient populations [9,10]. In a head-to-head phase 3 study, zanubrutinib was the only BTKi to demonstrate superiority over ibrutinib in patients with R/R CLL/SLL [10].

Although several clinical trials and real-world studies have reported promising response rates with zanubrutinib in both tTN and R/R CLL populations, the available evidence remains fragmented. To date, no comprehensive evidence synthesis has pooled efficacy outcomes across diverse study designs to provide a robust estimate of zanubrutinib’s clinical benefit. In particular, differences in treatment settings (TN vs. R/R), response categories, and patient risk profiles highlight the need for a systematic evaluation. This gap in consolidated knowledge limits clinicians’ ability to contextualize zanubrutinib’s role in CLL management fully and to compare its benefit–risk profile against other BTKis.

The aim of this systematic review and single-arm meta-analysis is to synthesize and pool the available clinical evidence on the efficacy of zanubrutinib in patients with CLL, including both TN and R/R populations. The present study was designed as a single-arm meta-analysis to comprehensively evaluate the pooled efficacy outcomes of zanubrutinib across all available clinical trials, including both early-phase and non-comparative studies. This approach enabled us to capture a broader evidence base, extending beyond head-to-head randomized trials. While we acknowledge the availability of direct comparative data such as the ALPINE trial (zanubrutinib vs. ibrutinib), our objective was not to perform a comparative meta-analysis but rather to provide an overall quantitative summary of zanubrutinib’s performance across diverse study populations and settings. The inclusion of comparative trials in a pooled single-arm framework ensured the consistent extraction of zanubrutinib-specific outcomes, thereby avoiding heterogeneity introduced by differing comparator arms or treatment protocols.

Specifically, we seek to estimate the pooled ORR, complete response (CR), partial response (PR), nodular partial response (nPR), stable disease (SD), and progressive disease (PD) rates associated with zanubrutinib. Additionally, we aim to compare efficacy outcomes across subgroups stratified by treatment setting (TN vs. R/R) and critically appraise the methodological quality of included studies to inform the strength of the available evidence.

## 2. Methods

### 2.1. Study Protocol and Registration

Following the Preferred Reporting Items for Systematic Reviews and Meta-Analysis (PRISMA) [14], we carried out this systematic review and meta-analysis. We followed the recommendations of Cochrane’s Handbook of Systematic Reviews of Interventions [15] at every turn. With the protocol ID CRD420251149403, we registered the protocol in PROSPERO.

### 2.2. Search Strategy and Data Collection

We searched the Web of Science (WoS), Scopus, PubMed, and Medline electronic databases. We searched every study released until August 2025. Details of the search approach are (zanubrutinib OR “BGB-3111”) AND (“chronic lymphocytic leukemia” OR “CLL” OR “small lymphocytic lymphoma” OR “SLL” OR “Leukemia, Lymphocytic, Chronic, B-Cell”).

In two screening processes, we evaluated every study retrieved for our eligibility requirements. Studies that satisfied our eligibility requirements are included. The references to the earlier reviews and the studies we included were manually screened. Two authors completed all screening, and a third reviewer settled all conflicts.

### 2.3. Eligibility Criteria

Given the heterogeneity of study designs, this review was conducted as a single-arm meta-analysis to estimate pooled efficacy outcomes (ORR, CR, and PR) of zanubrutinib across all available studies in CLL. Studies with or without comparator arms were eligible for inclusion, provided that efficacy data for the zanubrutinib arm could be independently extracted. For instance, data from the ALPINE trial—although a head-to-head comparison with ibrutinib—were incorporated by including only the zanubrutinib treatment arm to maintain analytical consistency. This approach aligns with PRISMA guidelines, which allow pooling of single-arm data when the objective is to summarize treatment efficacy rather than conduct comparative analyses.

We included RCTs that fulfilled the illustrated PICO criteria as follows: population (P): patients with CLL/SLL, either TN or R/R cases; intervention (I): zanubrutinib; outcome (O): efficacy outcomes; and study design (S): any clinical trials that assess the efficacy of zanubrutinib in patients with CLL/SLL.

### 2.4. Data Extraction and Outcome Measurement

Two authors used Excel sheets to extract data. A third author resolved the conflicts of data extraction.

### 2.5. Quality Assessment

The revised Joanna Briggs Institute (JBI) critical appraisal tool for the assessment of risk of bias for RCTs was used by two authors to evaluate the methodology of the included studies [16]. This validated tool evaluates methodological quality across 13 key domains, including randomization, allocation concealment, baseline comparability, blinding of participants, personnel, and outcome assessors, reliability of outcome measures, completeness of follow-up, and appropriate statistical analysis.

The methodological quality of the included non-RCTs was assessed using the Joanna Briggs Institute (JBI) critical appraisal checklist for case series [17]. This validated tool comprises 10 items that evaluate key domains of methodological rigor. Based on the information provided in the publication, each criterion was rated as “Yes” (criterion met), “No” (criterion not met), “Unclear,” or “Not applicable”.

Two independent reviewers performed the appraisal, and discrepancies were resolved by consensus with a third reviewer. The results of the quality appraisal were summarized in tabular form. Notably, studies were not excluded based on quality scores, but the findings of the assessment were considered during data synthesis and interpretation of results. 

### 2.6. Data Analysis

We performed a single-arm meta-analysis to estimate the pooled effect of zanubrutinib in patients with CLL, including both treatment-naïve and relapsed/refractory populations. Data synthesis and statistical analyses were conducted using Open MetaAnalyst 3.1 beta software.

For each study, we extracted the proportion of patients achieving the predefined outcomes (e.g., overall response rate [ORR], complete response [CR], partial response [PR], nodular partial response [nPR], stable disease [SD], and progressive disease [PD]). Standard errors were calculated from the reported proportions and sample sizes. When studies reported zero events in one or both arms, a continuity correction of 0.5 was applied to allow for the calculation of pooled estimates. When necessary, raw data were used to reconstruct proportions.

A random-effects model (DerSimonian and Laird method) was employed to account for potential clinical and methodological heterogeneity across studies. Pooled estimates were presented with 95% confidence intervals (CIs). Heterogeneity was quantified using the Cochran’s Q test (*p* < 0.10 considered significant) and the I^2^ statistic, with thresholds of 25%, 50%, and 75% indicating low, moderate, and high heterogeneity, respectively.

Subgroup analyses were planned according to treatment status (treatment-naïve vs. relapsed/refractory). Sensitivity analyses were performed by sequentially omitting individual studies to assess the robustness of the pooled estimates.

Publication bias was not formally assessed, as fewer than ten studies were included in the pooled analyses, which is below the threshold recommended for reliable evaluation using funnel plots or statistical tests such as Egger’s or Begg’s tests.

## 3. Results

### 3.1. Study Selection

**Figure 1:**
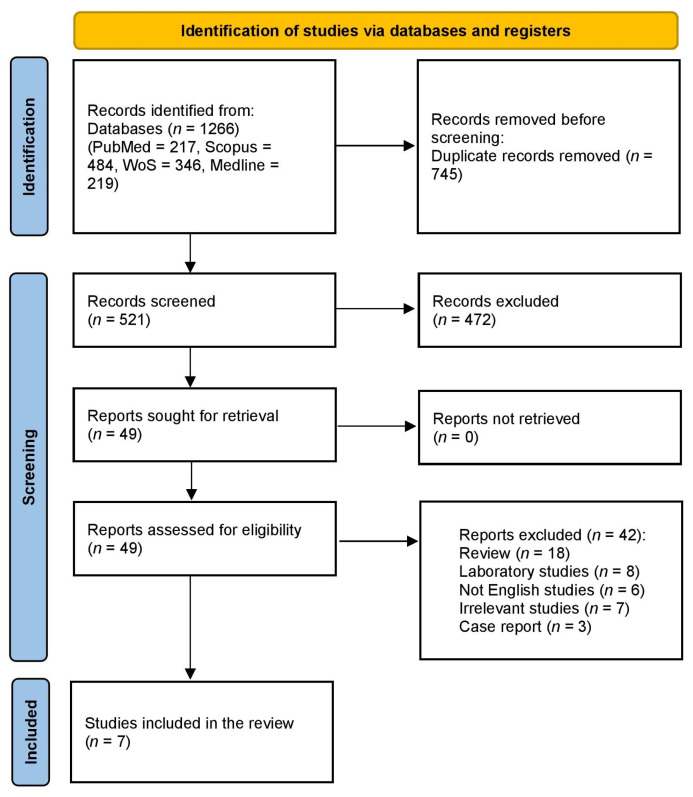
PRISMA flowchart of the included studies.

Database searches identified 1266 records. After removing duplicates, 521 studies were screened during the title and abstract screening. Full-text screening was conducted for 49 studies, of which 42 were excluded. Ultimately, seven studies met the inclusion criteria and were included in this review. More details are presented in Figure 1.

### 3.2. Study Characteristics

Seven studies met the eligibility criteria, comprising both clinical trials and real-world observational research [9,10,13,18,19,20,21]. These studies included patients with CLL or SLL who were either TN or had R/R disease. Table 1 summarizes the baseline characteristics of the included studies. The included studies consisted of two phase III RCTs [9,10], four phase I/II or II single-arm studies [13,18,20,21], and one multicenter real-world retrospective study [19]. The trials were conducted across diverse geographic regions, including Asia, North America, Europe, and Oceania, highlighting the global applicability of zanubrutinib data.

Most trials were prospectively registered on ClinicalTrials.gov (e.g., NCT04172246, NCT04116437, NCT03734016, NCT03336333, NCT02343120, NCT03206918), while the real-world study did not have formal registration. Study durations ranged from approximately 9 months [21] to over 4 years [20], with median follow-up periods ranging from 15.1 to 47.2 months.

The total number of patients treated with zanubrutinib across studies exceeded 1000. Sample sizes varied from 19 patients in the small Japanese phase I/II trial [13] to 327 patients in the international phase III ALPINE trial [10]. Three studies included both TN and R/R patients [13,19,20], while three studies focused specifically on R/R cohorts [10,18,21] and only one study included TN populations with high comorbidity burden [9]. Notably, Shadman and colleagues enrolled patients intolerant to prior BTKi (ibrutinib or acalabrutinib), offering insights into the tolerability of zanubrutinib in this unique group [18].

Diagnosis was established using internationally recognized criteria, most commonly the International Workshop on Chronic Lymphocytic Leukemia (iwCLL) 2008 or 2018 guidelines [22]. The WHO classification for SLL and the Lugano criteria were also used where appropriate [23]. All studies administered zanubrutinib orally, with the standard regimen being 160 mg twice daily. Some protocols also allowed 320 mg once daily as an alternative schedule [18,20]. Treatment was given continuously in 28-day cycles until disease progression, unacceptable toxicity, or withdrawal. More details were presented in Table 1.

### 3.3. Patient Characteristics

The median age across studies ranged from 61 years [10] to 71 years [4,5], and most cohorts were predominantly male (female proportion 25–49%).

Eastern Cooperative Oncology Group (ECOG) performance status (PS) was generally favorable. In most studies, the majority of patients had an ECOG PS of 0–1. All studies included patients with either CLL or SLL, with CLL comprising the majority (≥90% in most cohorts). Binet stage at diagnosis was variably reported, with studies showing distribution across stages A–C. Both TN and R/R populations were represented. Tam et al. (2022) exclusively enrolled TN patients [9], while Shadman et al. (2025) and Xu et al. (2020) included only R/R patients [10,18,21]. Mixed populations were reported by three studies [13,19,20], with R/R patients generally comprising the majority.

Reporting of baseline cytopenia varied. In Cull et al. (2021), 53.7% had at least one cytopenia (hemoglobin ≤ 110 g/L, platelet count ≤ 100 × 10^9^/L, or ANC ≤ 1.5 × 10^9^/L) [20], whereas Shadman et al. (2025) observed lower frequencies (15.5% with anemia, 8.5% with thrombocytopenia, and 4.2% with neutropenia) [18]. Xu et al. (2020) noted that 27.5% had neutropenia at baseline [21].

High-risk genomic abnormalities were frequent. TP53 disruption (del[17p] and/or TP53 mutation) ranged from 8.5% in [18] to 31.8% [9]. del(11q) was reported in 10.7–27.8% of patients, while del(13q) and trisomy 12 were also common (up to 59.7% and 23.1%, respectively). Complex karyotype (≥3 abnormalities) was reported in 7.9–17.1% of patients. IGHV mutational status was inconsistently reported, with unmutated disease predominating (43–73%).

Bulky lymphadenopathy (≥5 cm) was present in 14–44% of patients across studies. Two studies provided more detailed distributions, with up to 4.1% having very bulky disease (≥10 cm) [20,21]. More details are presented in Table 2.

### 3.4. Quality Assessment

Seven studies, including five non-randomized studies and two RCTs, were assessed for methodological quality [9,10].

Overall, the included non-randomized studies demonstrated moderate-to-high methodological quality. Most studies clearly defined inclusion criteria, used reliable diagnostic methods, and reported outcomes and follow-up data consistently. Demographic and clinical characteristics of participants were generally well documented. All studies applied standard diagnostic criteria (iwCLL or WHO) and used appropriate statistical methods. Clinical outcomes and follow-up results were consistently reported. In contrast, some studies did not explicitly clarify whether participant inclusion was consecutive [13,19]. More details are presented in Table 3.

The two RCTs [9,10] were generally of good methodological quality, with adequate randomization procedures and appropriate statistical analyses. Brown and collogues reported apparent randomization, concealed allocation, and comparable baseline characteristics between treatment groups [10]. Outcomes were consistently measured, and follow-up was complete. However, participants and treating physicians were not blinded to treatment assignment, which may introduce performance bias. Tam and colleagues also used randomization but had unclear allocation concealment and unclear blinding of outcome assessors. In addition, treatment groups were not treated identically beyond the intervention, which may have influenced results [9]. Both RCTs maintained reliable outcome measurement and appropriate statistical analysis, ensuring overall validity of findings despite limitations in blinding. More details are presented in Table 4.

### 3.5. Primary and Secondary Outcomes

#### 3.5.1. Overall Response Rate (ORR) in Combined (TN & R/R) Patients

**Figure 2:**
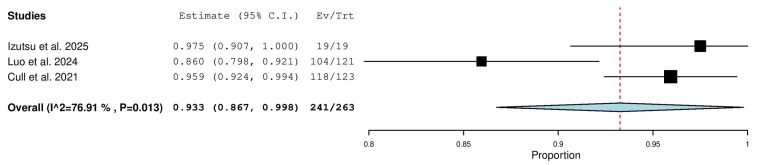
Overall Response Rate (ORR) in Combined (TN & R/R) Patients [13,19,20].

Three studies, including a total of 263 patients with CLL (both TN and R/R), reported on the overall response rate. The pooled analysis yielded an ORR of 93.3% (95% CI, 86.7–99.8%), with significant heterogeneity observed across studies (I^2^ = 76.91%, *p* = 0.013). In total, 241 of 263 patients responded to treatment (Figure 2).

#### 3.5.2. Overall Response Rate (ORR) in Relapsed/Refractory (R/R) Patients

**Figure 3:**
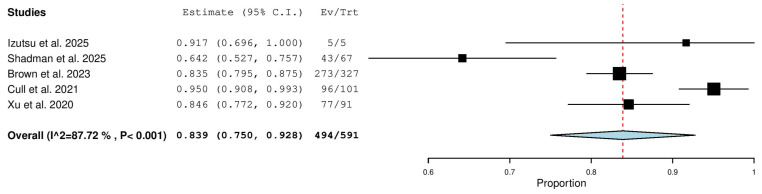
Overall Response Rate (ORR) in Relapsed/Refractory (R/R) Patients [10,13,18,20,21].

Five studies, including a total of 591 patients with R/R CLL, reported on the overall response rate. The pooled analysis yielded an ORR of 83.9% (95% CI, 75.0–92.8%), with significant heterogeneity observed across studies (I^2^ = 87.72%, *p* < 0.001). In total, 494 of 591 patients responded to treatment (Figure 3).

#### 3.5.3. Overall Response Rate (ORR) in Treatment-Naïve (TN) Patients

**Figure 4:**
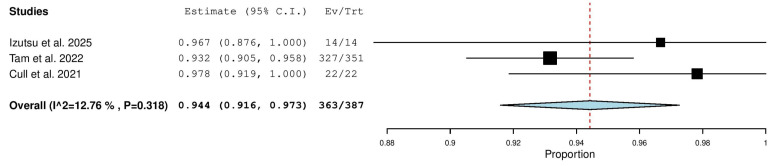
Overall Response Rate (ORR) in Treatment-Naïve (TN) Patients [9,13,20].

Three studies, including a total of 387 patients with TN CLL, reported on the overall response rate. The pooled analysis yielded an ORR of 94.4% (95% CI, 91.6–97.3%), with low heterogeneity observed across studies (I^2^ = 12.76%, *p* = 0.318). In total, 363 of 387 patients responded to treatment (Figure 4).

#### 3.5.4. Complete Response (CR) Rate in Combined (TN & R/R) Patients

**Figure 5:**
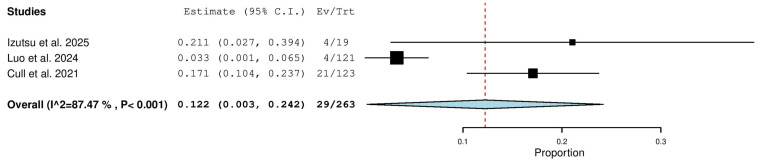
Complete Response (CR) Rate in Combined (TN & R/R) Patients [13,19,20].

Three studies, including a total of 263 patients with CLL (both TN and R/R), reported on the rate of complete response with zanubrutinib therapy. The pooled analysis yielded a complete response rate of 12.2% (95% CI, 0.3–24.2%), with significant heterogeneity observed across studies (I^2^ = 87.47%, *p* < 0.001). In total, 29 of 263 patients achieved a complete response. The heterogeneity may reflect differences in patient populations (treatment-naive vs. relapsed/refractory), study design, and response assessment criteria (Figure 5).

#### 3.5.5. Complete Response (CR) Rate in Relapsed/Refractory (R/R) Patients

**Figure 6:**
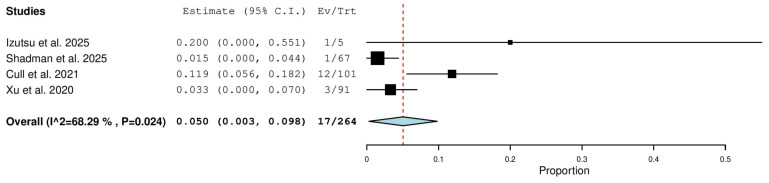
Complete Response (CR) Rate in Relapsed/Refractory (R/R) Patients [13,18,20,21].

Four studies, including 264 patients with R/R CLL, reported on the rate of complete response with zanubrutinib therapy. The pooled analysis yielded a complete response rate of 5.0% (95% CI, 0.3–9.8%), with significant heterogeneity observed across studies (I^2^ = 68.29%, *p* = 0.024). In total, 17 of 264 patients achieved a complete response (Figure 6).

#### 3.5.6. Complete Response (CR) Rate in Treatment-Naïve (TN) Patients

**Figure 7:**
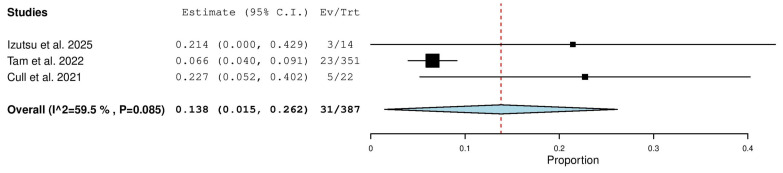
Complete Response (CR) Rate in Treatment-Naïve (TN) Patients [9,13,20].

Three studies, including a total of 387 patients with TN CLL, reported on the rate of complete response with zanubrutinib therapy. The pooled analysis yielded a complete response rate of 13.8% (95% CI, 1.5–26.2%), with moderate heterogeneity observed across studies (I^2^ = 59.5%, *p* = 0.085). In total, 31 of 387 patients achieved a complete response (Figure 7).

#### 3.5.7. Nodular Partial Response (nPR) Rate in Relapsed/Refractory (R/R) Patients

**Figure 8:**
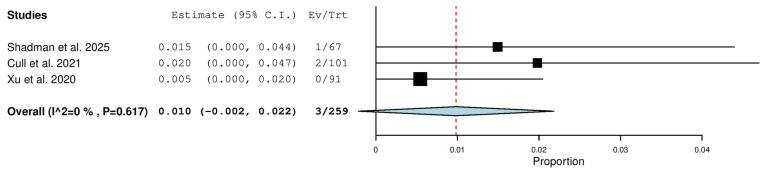
Nodular Partial Response (nPR) Rate in Relapsed/Refractory (R/R) Patients [18,20,21].

Three studies, including a total of 259 patients with R/R CLL, reported on the rate of nodular partial response. The pooled analysis yielded an nPR rate of 1.0% (95% CI, −0.2–2.2%), with no significant heterogeneity observed across studies (I^2^ = 0%, *p* = 0.617). In total, three of 259 patients achieved an nPR (Figure 8).

#### 3.5.8. Partial Response (PR) Rate in Combined (TN & R/R) Patients

**Figure 9:**
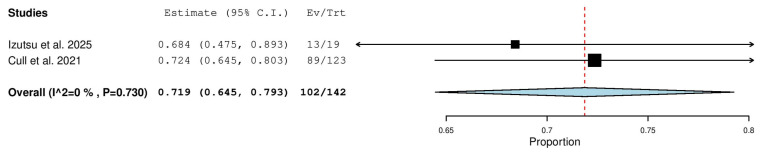
Partial Response (PR) Rate in Combined (TN & R/R) Patients [13,20].

Two studies, including a total of 142 patients with CLL (both TN and R/R), reported on the rate of partial response. The pooled analysis yielded a PR rate of 71.9% (95% CI, 64.5–79.3%), with no significant heterogeneity observed across studies (I^2^ = 0%, *p* = 0.730). In total, 102 of 142 patients achieved a partial response (Figure 9).

#### 3.5.9. Partial Response (PR) in Relapsed/Refractory (R/R) Patients

**Figure 10:**
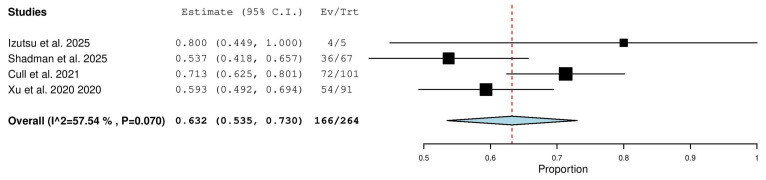
Partial Response (PR) Rate in Relapsed/Refractory (R/R) Patients [13,18,20,21].

Four studies, including a total of 264 patients with R/R CLL, reported on the rate of partial response. The pooled analysis yielded a PR rate of 63.2% (95% CI, 53.5–73.0%), with moderate heterogeneity observed across studies (I^2^ = 57.54%, *p* = 0.070). In total, 166 of 264 patients achieved a partial response (Figure 10).

#### 3.5.10. Partial Response (PR) in Treatment-Naïve (TN) Patients

**Figure 11:**
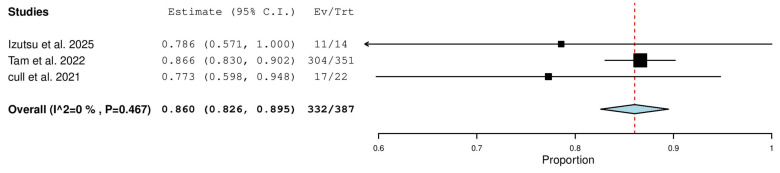
Partial Response (PR) Rate in Treatment-Naïve (TN) Patients [9,13,20].

Three studies, including a total of 387 patients with TN CLL, reported on the rate of partial response. The pooled analysis yielded a PR rate of 86.0% (95% CI, 82.6–89.5%), with no significant heterogeneity observed across studies (I^2^ = 0%, *p* = 0.467). In total, 332 of 387 patients achieved a partial response (Figure 11).

#### 3.5.11. Progressive Disease (PD) Rate in Combined (TN & R/R) Patients

**Figure 12:**
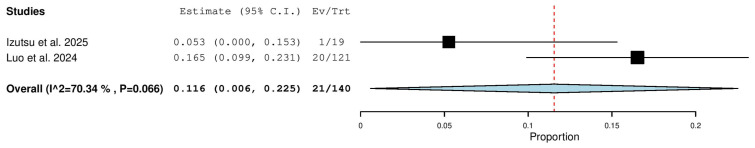
Progressive Disease (PD) Rate in Combined (TN & R/R) Patients [13,19].

Two studies, including a total of 140 patients with CLL (both TN and R/R), reported on the rate of progressive disease. The pooled analysis yielded a PD rate of 11.6% (95% CI, 0.6–22.5%), with considerable heterogeneity observed across studies (I^2^ = 70.34%, *p* = 0.066). In total, 21 of 140 patients had progressive disease (Figure 12).

#### 3.5.12. Progressive Disease (PD) Rate in Relapsed/Refractory (R/R) Patients

**Figure 13:**
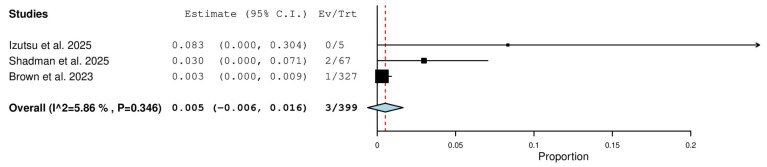
Progressive Disease (PD) Rate in Relapsed/Refractory (R/R) Patients [10,13,18].

Three studies, including a total of 399 patients with R/R CLL, reported on the rate of progressive disease. The pooled analysis yielded a PD rate of 0.5% (95% CI, −0.6–1.6%), with no significant heterogeneity observed across studies (I^2^ = 5.86%, *p* = 0.346). In total, 3 of 399 patients had progressive disease (Figure 13).

#### 3.5.13. Stable Disease (SD) Rate in Relapsed/Refractory (R/R) Patients

**Figure 14:**
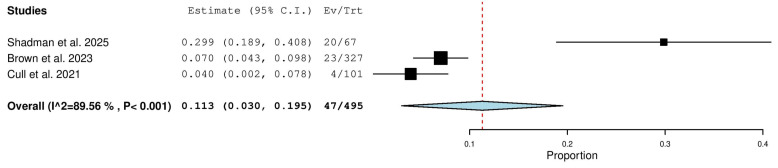
Stable Disease (SD) Rate in Relapsed/Refractory (R/R) Patients [10,18,20].

Three studies, including a total of 495 patients with R/R CLL, reported on the rate of stable disease. The pooled analysis yielded an SD rate of 11.3% (95% CI, 3.0–19.5%), with significant heterogeneity observed across studies (I^2^ = 89.56%, *p* < 0.001). In total, 47 of 495 patients had stable disease (Figure 14).

## 4. Discussion

This systematic review and single-arm meta-analysis synthesized evidence from six clinical trials and one real-world study, evaluating the efficacy of zanubrutinib in patients with CLL, both TN and R/R. The pooled analyses demonstrated consistently high ORR, particularly in TN populations (94.4%), with robust responses also observed in R/R patients (83.9%). These findings highlight zanubrutinib as a highly effective BTKi across diverse clinical settings.

This meta-analysis synthesized data from both single-arm and comparative trials by extracting outcomes specific to the zanubrutinib treatment arms. While the ALPINE trial included a comparator (ibrutinib), its zanubrutinib-specific efficacy data were analyzed independently to provide a comprehensive assessment of the drug’s performance across diverse settings. The choice of a single-arm meta-analytic approach was based on the objective to derive pooled estimates of response rates and disease control associated with zanubrutinib, rather than to directly compare its efficacy to other BTKis.

This approach aligns with PRISMA recommendations for synthesizing heterogeneous evidence when comparative data are limited or inconsistent across studies. However, it also introduces inherent limitations, most notably, the absence of direct comparative inference and the potential for selection and reporting biases inherent in single-arm data. Future meta-analyses incorporating head-to-head comparative data (e.g., zanubrutinib vs. ibrutinib or acalabrutinib) would be valuable to further contextualize relative efficacy and safety.

### 4.1. Efficacy Findings

Notably, CR rates remained relatively modest, ranging from 5.0% in R/R populations to 13.8% in TN cohorts, reflecting the well-recognized challenge of achieving deep remission with BTKi. The higher PR rates, particularly in TN patients (86.0%), reinforce the role of zanubrutinib in inducing durable disease control even if complete eradication of disease is uncommon. Notably, the low rates of PD in both TN and R/R cohorts highlight its effectiveness in maintaining disease stability over extended follow-up.

These results align with and extend findings from pivotal RCTs, including the ALPINE and SEQUOIA trials, which established zanubrutinib as a preferred alternative to ibrutinib and acalabrutinib due to improved tolerability and comparable or superior efficacy. Our pooled estimates provide further quantitative support that zanubrutinib achieves high disease control rates across heterogeneous patient populations, including those intolerant to prior BTKi.

However, considerable heterogeneity was observed in some analyses, particularly for CR and SD rates. This may reflect differences in baseline patient risk profiles (e.g., TP53 disruption, IGHV status, bulky disease), trial designs, and response assessment criteria. The real-world evidence also highlighted variability in patient characteristics and dosing practices, which may influence outcomes compared with controlled trial settings.

In a head-to-head phase 3 study, zanubrutinib was the only BTKi to demonstrate superiority over ibrutinib in patients with R/R CLL/SLL [10]. In Izutsu et al. (2025) [13], Japanese patients’ ECOG PS was much lower than that of patients in international studies (ECOG PS of 0: 100% of Japanese patients versus 39.4% of patients with R/R CLL/SLL in ALPINE [10]; 85.7% of Japanese patients with TN CLL/SLL versus 45.6% of patients in SEQUOIA) [5]. Del(17p) was absent in Japanese patients with CLL/SLL in BGB-3111-111, and it was present in 13.8% of patients with R/R CLL/SLL in ALPINE [10], and SEQUOIA contained distinct cohorts of patients with and without del(17p) [9]. Japanese patients may have better results than participants in international research since they have a lower prevalence of risk factors. When compared to the first-generation BTKi ibrutinib, zanubrutinib showed a better ORR and progression-free survival (PFS) in patients with R/R CLL/SLL in the head-to-head phase 3 ALPINE study [10,24]. In the primary goal of investigator-assessed ORR (PR or better, 78.3% versus 62.5%, respectively), zanubrutinib outperformed ibrutinib in a planned interim analysis of the study (median follow-up, 15 months) [24]. Later, zanubrutinib continued to show an improved ORR (83.5% versus 74.2% in patients treated with ibrutinib) in the predetermined final analysis of PFS (median study follow-up, 29.6 months) and outperformed ibrutinib in the crucial secondary endpoint of PFS (hazard ratio [HR], 0.65; 95% CI, 0.49–0.86; *p* = 0.002) [10].

Longer follow-up (median trial follow-up, 42.5 months) produced consistent results, with zanubrutinib outperforming ibrutinib in terms of PFS across subgroups with high-risk characteristics [25]. Japanese patients with R/R CLL/SLL exhibited comparable outcomes, with a 100% ORR and 100% PFS rate at 12 and 24 months [13]. With a median follow-up of 17.7 months (100% in R/R CLL/SLL by investigator), the ORR was high at the data cut-off of May 10, 2022, and it has gotten deeper over time. Regardless of the presence of a del(17p) mutation, zanubrutinib was demonstrated to be highly effective in patients with TN CLL/SLL in the phase 3 SEQUOIA trial. At a median study follow-up of 26.2 months, it significantly improved PFS when compared to bendamustine and rituximab (BR) (HR, 0.42; 95% CI, 0.28–0.63; *p* < 0.0001) [9]. Investigator-assessed PFS at 24 months was similar in patients with del(17p) treated with zanubrutinib (87.0%) and greater in patients without del(17p) treated with zanubrutinib (87.7%) compared to patients treated with BR (76.5%) in SEQUOIA [9]. In patients without del(17p), investigator-assessed ORR (PR-L or greater) was 97.5% with zanubrutinib, compared to 88.7% with BR, and 96.4% with zanubrutinib in patients with del(17p) [9]. In contrast, the event-free rate of 85.7% at 12 months and 71.4% at 24 months for Japanese patients with TN CLL/SLL in BGB-3111-111 was 100%, the ORR was 100%, and the median PFS was not met; these results were in line with those in SEQUOIA. CR rates for Japanese and international patients with TN CLL/SLL who were enrolled in SEQUOIA were 21% and 9%, respectively [9]. CR rates for patients with R/R CLL/SLL who were enrolled in ALPINE were 20% for Japanese patients and 7% for patients from other countries [10].

### 4.2. Sources of Heterogeneity

Substantial heterogeneity was observed in the pooled estimates of ORR among R/R cohorts and in CR rates (I^2^ > 75%). This variability is likely multifactorial. Differences in baseline patient characteristics, including disease stage, prior lines of therapy, and risk profiles (e.g., TP53 mutation or IGHV unmutated status), may have contributed to the observed inconsistency. In addition, variation in follow-up duration, sample size, and response assessment criteria (such as investigator-assessed versus independent review) across the included studies may have influenced response estimates. Although subgroup analyses based on genomic markers could have provided deeper insight into these differences, the available data were insufficient for a reliable quantitative synthesis. Therefore, the observed heterogeneity should be interpreted with caution, and future studies with standardized response criteria and detailed molecular profiling are needed to clarify the impact of patient- and study-level factors on treatment outcomes.

The inclusion of a real-world study, Luo and colleagues, alongside clinical trial data may have contributed to the observed heterogeneity in pooled outcomes [19]. Real-world studies often include more diverse patient populations, broader eligibility criteria, and variable treatment adherence compared with controlled clinical trials. These differences can influence response rate outcomes, leading to increased variability across studies. Nevertheless, incorporating real-world evidence provides valuable insight into zanubrutinib’s performance in routine clinical practice and enhances the external validity of the findings. Although a separate quantitative analysis of real-world versus trial data was not feasible due to the limited number of studies, future meta-analyses with a larger evidence base could more clearly delineate the impact of real-world data on pooled efficacy and safety estimates.

### 4.3. Strengths and Limitations

This review represents the first attempt to quantitatively synthesize efficacy outcomes of zanubrutinib in both TN and R/R CLL patients. By including data from both RCTs and real-world studies, the analysis enhances the generalizability of findings across a wide range of clinical practice settings. The methodological rigor of this review further strengthens its validity; the study was prospectively registered on PROSPERO, a comprehensive and systematic search strategy was employed across multiple databases, and duplicate screening with standardized data extraction ensured consistency and minimized bias. Additionally, the use of random-effects meta-analysis allowed for the consideration of both methodological and clinical heterogeneity across included studies. Subgroup analyses by treatment status provided further clinically meaningful insights, distinguishing the efficacy of zanubrutinib between TN and R/R populations.

Despite these strengths, several limitations should be acknowledged. The number of included studies was modest, and some trials, such as the early-phase Japanese study, had small sample sizes that limited statistical power.

It is important to note that several of the included studies were based on relatively small patient cohorts. The limited sample sizes in these studies may reduce the statistical power and precision of the pooled estimates, potentially contributing to variability in the overall results. Small studies are also more susceptible to random error and publication bias, which may influence the observed treatment effects. Nevertheless, their inclusion was justified to ensure a comprehensive synthesis of all available evidence on zanubrutinib, especially in disease subgroups where larger randomized trials remain scarce. Future large-scale, multicenter studies are warranted to validate these findings and provide more robust estimates of efficacy and safety outcomes.

Another important limitation of this meta-analysis is the lack of access to individual patient data (IPD), which restricted our ability to adjust for potential confounding variables such as baseline disease characteristics, prior therapies, comorbidities, and molecular risk factors. As a result, the pooled estimates represent aggregate-level findings and may not fully account for between-study differences in patient populations or treatment settings. Access to IPD would enable more detailed subgroup and multivariable analyses, allowing for more accurate assessment of zanubrutinib’s efficacy and safety across clinically relevant subgroups. Future IPD-based meta-analyses are therefore warranted to overcome these limitations and to provide more refined, patient-level insights.

Potential publication bias represents another limitation of this meta-analysis. Studies with negative or non-significant findings may be less likely to be published, potentially leading to an overestimation of treatment efficacy in pooled analyses. Although the assessment of publication bias using funnel plots or formal statistical tests (e.g., Egger’s or Begg’s tests) is commonly recommended, such analyses are considered unreliable when fewer than ten studies are included. Given that the present meta-analysis did not meet this threshold, we did not perform a formal funnel plot analysis. Nevertheless, the possibility of publication bias cannot be excluded and should be considered when interpreting the results.

The absence of a quantitative safety meta-analysis represents an additional limitation of this study. This omission primarily reflects the inconsistent and incomplete reporting of adverse events across the included studies. Although several trials described common treatment-related events such as atrial fibrillation, bleeding, neutropenia, and infections, the variability in definitions, grading systems, and reporting formats made it difficult to pool data reliably. Consequently, a formal meta-analysis of safety outcomes was not feasible. Nonetheless, the available evidence suggests that zanubrutinib is generally well tolerated, with a lower incidence of certain cardiovascular and bleeding events compared to first-generation BTKi. Future studies should aim for standardized and comprehensive safety reporting to enable more robust quantitative synthesis of adverse event profiles.

An additional source of potential bias in this meta-analysis arises from the reliance on single-arm data without direct comparators. The absence of control or reference groups limits the ability to contextualize the efficacy outcomes of zanubrutinib relative to other BTKis. Differences in study design, patient selection, disease stage, prior treatments, and follow-up duration across individual studies may further influence pooled response rates and progression outcomes. Consequently, the findings should be interpreted as descriptive rather than comparative, providing an overall summary of zanubrutinib’s performance rather than evidence of superiority or equivalence to other agents such as ibrutinib or acalabrutinib. Future randomized or network meta-analyses are warranted to more accurately define zanubrutinib’s relative efficacy, safety, and clinical positioning among BTKis.

Furthermore, variations in follow-up duration across studies could have influenced the detection of responses and progression events, introducing potential bias into pooled estimates. Another limitation was the lack of individual patient-level data, which prevented the exploration of prognostic biomarkers, such as TP53 disruption and IGHV mutation status, as modifiers of response. Finally, safety outcomes were not systematically synthesized in this review, limiting the ability to draw firm conclusions regarding the overall benefit–risk balance of zanubrutinib.

### 4.4. Clinical Implications

The findings of this review reinforce zanubrutinib as an effective therapeutic option for patients with CLL across both TN and R/R populations. The consistently high ORR and durable disease control observed highlight its clinical value. Notably, the higher efficacy demonstrated in TN patients highlights zanubrutinib’s potential role as a frontline therapy, particularly in individuals with significant comorbidities who may not tolerate chemoimmunotherapy. In the R/R setting, the low progression rates, including among patients previously intolerant to other BTKis, support its role as a valuable salvage treatment. Collectively, these results provide clinicians with evidence-based guidance for treatment decision-making and offer quantitative benchmarks for ongoing and future head-to-head trials, as well as real-world comparative studies of BTKis.

## 5. Conclusions

This systematic review and single-arm meta-analysis demonstrated that zanubrutinib achieves high ORR in both TN and R/R CLL, with particularly strong efficacy in the frontline setting. Although complete responses remain relatively uncommon, the durability of PR and low progression rates highlight zanubrutinib’s potency as a disease-controlling therapy. Compared with first-generation BTKis such as ibrutinib, zanubrutinib offers a more favorable tolerability profile and comparable efficacy to second-generation agents like acalabrutinib, as supported by emerging head-to-head data. Clinically, zanubrutinib appears to be a valuable treatment option across both TN and R/R populations, including patients with high-risk genomic features such as TP53 mutations or IGHV unmutated status. Future research should focus on longer-term survival outcomes, patient-reported quality-of-life measures, and comparative analyses to further define zanubrutinib’s optimal role within the evolving landscape of BTK inhibition in CLL management.

## Figures and Tables

**Table 1:** Summary of the included studies.

Study ID	Type of Study	Country	Duration	Trial Registration	Type of Patients	Diagnostic Criteria	Zanubrutinib Regimen (Dose, Route of Administration, Regimen)	No. of Patients in the Zanubrutinib Group	Follow-Up, Months, Median (Range)
**Izutsu et al., 2025** [13]	Phase I/II, multicenter, open-label study	Japan	From 30 January 2020 to 31 October 2022.	NCT04172246	Japanese adults (age ≥ 20 years) with TN or R/R CLL/SLL or WM. Also included patients with other B-cell malignancies in Part 1 and an R/R MCL cohort in Part 2 for safety analysis.	CLL/SLL: 2018 iwCLL guidelines; SLL: Lugano classification; WM: 6th International Workshop on WM criteria	Dose: 160 mg twice daily Route: orally Regimen: continuously until disease progression, unacceptable toxicity, or other discontinuation criteria	CLL/SLL: 19 (TN: 14, R/R: 5)	27.9
**Shadman et al., 2025** [18]	Phase II, single-arm, open-label clinical trial	United States	Data Cut-off: 1 May 2024	NCT04116437	Patients with CLL or SLL who are intolerant of prior ibrutinib and/or acalabrutinib therapy	iwCLL criteria	Dose: 160 mg twice daily or 320 mg once daily Route: Oral (implied) Regimen: Continuous dosing; patients/investigators selected a regimen and could not switch	71	34.5 (0.1–51.1)
**Luo et al., 2024** [19]	Multicenter, real-world, retrospective observational study	China	The last follow-up time was 15 September 2024.	NR (real-world study)	Adult patients with CLL/SLL. TN: 90 patients (65.2%) R/R: 48 patients (34.8%)	iwCLL 2018 guidelines.	Standard Dose: 160 mg twice daily (orally), dose reduction to 80 mg twice daily	138	36.8 (95% CI: 34.5–39.1)
**Brown et al., 2023** [10]	Phase III, randomized, open-label, active-controlled, multinational, clinical Trial	Multinational (15 countries across North America, Europe, and the Asia Pacific)	From 1 November 2018, through 14 December 2020	NCT03734016	Relapsed or refractory CLL or SLL	iwCLL criteria	Dose: 160 mg twice daily Route: orally Regimen: until disease progression or unacceptable toxicity	327 patients in the zanubrutinib group (intention-to-treat population)	29.6
**Tam et al., 2022** [9]	Randomized, controlled, phase III trial	14 countries and regions (multicenter)	From 31 October 2017 to 22 July 2019	NCT03336333	Patients aged ≥65 years, or ≥18 years with comorbidities, with untreated CLL or SLL requiring treatment per iwCLL criteria	iwCLL criteria	Dose: 160 mg twice per day Route: Orally Regimen: in 28-day cycles until disease progression or unacceptable toxicity	Group A (without del(17p)): 241, Group C (with del(17p)): 111	26.2 (IQR 23·7–29·6)
**Cull et al., 2021** [20]	Phase I/II, open-label, single-arm study	Multinational: Australia, USA, New Zealand, China	From September 2014 to November 2018 (last first dose) Data cut-off: March 2021 (study closure)	NCT02343120	TN CLL/SLL: 22 patients (17.9%) R/R CLL/SLL: 101 patients (82.1%)	CLL/SLL diagnosed according to the WHO classifications. Requirement for treatment per the iwCLL criteria.	Dose: 160 mg twice daily (81 patients) or 320 mg once daily (40 patients). Two patients received 160 mg once daily (in the initial dose escalation phase). Route: Orally Regimen: Administered in 28-day cycles until disease progression or unacceptable toxicity.	Total: 123 patients (118 CLL, 5 SLL)	47.2
**Xu et al., 2020** [21]	Phase II, single-arm, multicenter study	China	From 9 March to 14 December 2017	CTR20160890 (7 December 2016) NCT03206918 (2 July 2017)	R/R CLL or SLL	CLL: iwCLL guidelines (2008); SLL: WHO classification (2008), histologically confirmed by central pathologic review	160 mg orally twice daily in 28-day cycles until disease progression or intolerance	91	15.1 (0.8–21.2)

**Abbreviations:** TN: treatment-naive, R/R: relapsed/refractory, CLL: chronic lymphocytic leukemia, SLL: small lymphocytic lymphoma, WM: Waldenström macroglobulinemia, MCL: mantle cell lymphoma, iwCLL: International Workshop on Chronic Lymphocytic Leukemia, WHO: World Health Organization.

**Table 2:** Baseline characteristics of the included patients.

Study ID	Age, Median (range)	Female n (%)	ECOG PS, No. (%)	Type of Cancer No. (%)	Binet Stage at CLL Diagnosis, n (%)	Prior Treatment Status No. (%)	Cytopenia at Baseline, No. (%)	Chromosomal Mutation Status, No. (%)	IGHV Mutational Status—No. (%)	Bulky Disease—No. (%)¶
**Izutsu et al.,** **2025** [13]	71.0 (38–77)	5 (26.3)	0: 17 (89.5) 1: 2 (10.5)	CLL/SLL: 19 (100)	NR	TN: 14 (73.7%) R/R: 5 (26.3%) R/R CLL/SLL (n = 5)	NR	CLL/SLL: del(17p): 0 (0)	Mutated: 12 (63.2) Unmutated: 7 (36.8)	NR
**Shadman et al., 2025** [18]	71 (49–91)	35 (49.3)	0: 44 (62) 1: 25 (35.2) 2: 2 (2.8)	CLL: 63 (88.7) SLL: 8 (11.3)	A: 12 (16.9) B: 6 (8.5) C: 4 (5.6) Unknown: 41 (57.7)	R/R: 71 (100%)	Hemoglobin ≤ 110 g/L: 11 (15.5) Platelet count ≤ 100 × 10^9^/L: 6 (8.5) ANC ≤ 1.5 × 10^9^/L: 3 (4.2)	del(11q): 10 (14.1) del(17p): 6 (8.5) del(13q): 17 (23.9) TP53 mutation: 16 (22.5)	Unmutated: 13 (18.3) Mutated: 12 (16.9) Unknown/Missing: 46 (64.8)	Largest diameter < 5 cm: 49 (69) Largest diameter > 5 cm: 10 (14.1) No measurable disease: 12 (16.9)
**Luo et al., 2024** [19]	68 (IQR: 37–87)	46 (33.3)	NR	CLL: 132 (95.7) SLL: 6 (4.3)	A: 18 (13.6) B: 55 (41.7) C: 59 (44.7)	TN: 90 (65.2) R/R: 48 (34.8)	NR	TP53 status (deletion and/or mutation): 27/109 (24.8) del(11q): 9/84 (10.7) del(17p): 15/105 (14.3) Complex Karyotype (≥3 abnormalities): 6/76 (7.9)	Unmutated IGHV: 29/67 (43.3)	NR
**Brown et al., 2023** [10]	67 (35–90) ≥65 and <75: 127 (38.8) ≥75: 74 (22.6)	114 (34.9)	≥1: 198 (60.6)	CLL or SLL: 327 (100)	A/B: 182 (55.7) C: 145 (44.3)	1 line: Zanubrutinib 192 (58.7) 2 lines: 86 (26.3) 3 lines: 25 (7.6) 3 lines: Zanubrutinib 24 (7.3)	NR	17p deletion and/or TP53 mutation: 75 (22.9) 11q deletion: Zanubrutinib 91 (27.8%), IComplex karyotype (≥3 abnormalities): 56 (17.1)	Unmutated: 239 (73.1) Mutated: 79 (24.2) Missing:9 (2.8)	Yes (tumor ≥ 5 cm): 145 (44.3)
**Tam et al., 2022** [9]	70 (IQR: 66–74)	119 (33.8)	0: 154/352 (43.8) 1: 169/352 (48.0) 2: 29/352 (8.2)	CLL: 321/352 (91.2) SLL: 31/352 (8.8)	A/B: 243 (69) C: 109 (31)	TN: 352 (100)	163 (46.4)	del(17p): 112 (31.8) del(11q): 80 (22.7) del(13q): 210 (59.7) Trisomy 12: 65 (18.5) No FISH abnormalities: 56 (15.9)	Unmutated: 192 (57.0) Mutated: 145 (43.0)	Bulky disease (≥5 cm): 113 (32.1%)
**Cull et al., 2021** [20]	67 (24–87)	31 (25.2)	0: 57 (46.3) 1: 61 (49.6) 2: 5 (4.1)	CLL: 118 (95.9) SLL: 5 (4.1)	NR	TN: 22 (17.8) R/R: 101 (82.2) 1 line: 49 (39.8) ≥3 lines: 28 (22.76)	Hemoglobin ≤ 110 g/L OR platelet count ≤ 100 × 10^9^/L OR absolute neutrophil count ≤ 1.5 × 10^9^/L Present: 66 (53.7)	del(17p): 16/99 (16.2) TP53 mutation: 14/43 (32.6) del(11q): 23/98 (23.5) del(13q): 45/98 (45.9) Trisomy 12: 15/97 (15.5) Both del(17p) and TP53 mutation: 6/101 (5.9)	Unmutated: 29/42 (69.0%) Mutated: 13/42 (31.0)	Defined as: Any target lesion with the longest diameter ≥ 5 cm, Present: 47 patients (38.2) ≥10 cm: 5 patients (4.1)
**Xu et al., 2020** [21]	61 (35–87)	39 (42.9)	0/1: 88 (96.7) 2: 3 (3.3)	CLL: 82 (90.1) SLL: 9 (9.9)	A/B: 27 (32.9) C: 55 (67.1)	Refractory to last systemic therapy: 72 (79.1) Non-refractory: 19 (20.9) ≥2 prior lines: 45 (49.5)	Present: 66 (72.5) had a baseline serum ẞ2-microglobulin level > 3.5 mg/L Absent: 25 (27.5) had a baseline absolute neutrophil count < 1.5 × 10^9^/L.	del(17p) or TP53 mutation: 22 (24.2) del(11q): 20 (22.0) del(13q): 41 (45.1) Trisomy 12: 21 (23.1)	Unmutated: 51 (56.0) Mutated: 23 (25.3) Not available: 17 (18.7)	Present (≥1 lesion with LDI ≥ 5 cm): 40 (44.0) Absent: 51 (56.0)

**Abbreviations:** TN: treatment-naive, R/R: relapsed/refractory, CLL: chronic lymphocytic leukemia, SLL: small lymphocytic lymphoma, ECOG: Eastern Cooperative Oncology Group.

**Table 3:** Quality Assessment of Non-Randomized Trials by JBI Tool.

Study ID	Izutsu et al., 2025 [13]	Shadman et al., 2025 [18]	Luo et al. 2024 [19]	Cull et al. 2021 [20]	Xu et al., 2020 [21]
1. Were there clear criteria for inclusion in the case series?	Yes	Yes	Yes	Yes	Yes
2. Was the condition measured in a standard, reliable way for all participants included in the case series?	Yes	Yes	Yes	Yes	Yes
3. Were valid methods used for identification of the condition for all participants included in the case series?	Yes	Yes	Yes	Yes	Yes
4. Did the case series have consecutive inclusion of participants?	Unclear	Yes	Unclear	Yes	Yes
5. Did the case series have complete inclusion of participants?	Yes	Yes	Yes	Yes	Yes
6. Was there clear reporting of the demographics of the participants included in the study?	Yes	Yes	Yes	Yes	Yes
7. Was there clear reporting of clinical information of the participants?	Yes	Yes	Yes	Yes	Yes
8. Were the outcomes or follow-up results of cases clearly reported?	Yes	Yes	Yes	Yes	Yes
9. Was there clear reporting of the presenting sites’/clinics’ demographic information?	Unclear	Unclear	Unclear	Yes	Unclear
10. Was statistical analysis appropriate?	Yes	Yes	Yes	Yes	Yes

**Table 4:** Quality Assessment of Randomized Trials by JBI Tool.

Study ID	Brown et al., 2023 [10]	Tam et al., 2022 [9]
Was true randomization used for assignment of participants to treatment groups?	YES	YES
Was allocation to treatment groups concealed?	YES	UNCLEAR
Were treatment groups similar at the baseline?	YES	YES
Were participants blind to treatment assignment?	NO	NO
Were those delivering treatment blind to treatment assignment?	NO	NO
Were outcomes assessors blind to treatment assignment?	YES	UNCLEAR
Were treatment groups treated identically other than the intervention of interest?	YES	NO
Was follow up complete and if not, were differences between groups in terms of their follow up adequately described and analyzed?	YES	YES
Were participants analyzed in the groups to which they were randomized?	YES	YES
Were outcomes measured in the same way for treatment groups?	YES	YES
Were outcomes measured in a reliable way?	YES	YES
Was appropriate statistical analysis used?	YES	YES
Was the trial design appropriate, and any deviations from the standard RCT design (individual randomization, parallel groups) accounted for in the conduct and analysis of the trial?	YES	YES

## Data Availability

No new data were created or analysed in this study. Data sharing is not applicable to this article.
